# Structural elucidation, molecular docking, α-amylase and α-glucosidase inhibition studies of 5-amino-nicotinic acid derivatives

**DOI:** 10.1186/s13065-020-00695-1

**Published:** 2020-07-14

**Authors:** Muhammad Nawaz, Muhammad Taha, Faiza Qureshi, Nisar Ullah, Manikandan Selvaraj, Sumaira Shahzad, Sridevi Chigurupati, Abdul Waheed, Fadiah Ammar Almutairi

**Affiliations:** 1 Department of Nano-Medicine Research, Institute for Research and Medical Consultations (IRMC), Imam Abdulrahman Bin Faisal University, P.O. Box 1982, Dammam, 31441 Saudi Arabia; 2 Department of Clinical Pharmacy, Institute for Research and Medical Consultations (IRMC), Imam Abdulrahman Bin Faisal University, P.O. Box 1982, Dammam, 31441 Saudi Arabia; 3 Deanship of Scientific Research, Imam Abdulrahman Bin Faisal University, P.O. Box 1982, Dammam, 31441 Saudi Arabia; 4 Chemistry Department, King Fahd University of Petroleum & Minerals, Dhahran, 31261 Saudi Arabia; 5 School of Chemical Engineering, Monash University, Bandar Subway, 47500 Selangor Darul Ehsan, Malaysia; 6 School of Business Administration, College of International Education, Zhejiang Gongshang University, Hangzhou, China; 7 Department of Medicinal Chemistry and Pharmacognosy, College of Pharmacy, Qassim University, Buraidah, 52571 Saudi Arabia

**Keywords:** 5-Amino-nicotinic acid, Spectral studies, NMR, HR-MS, FTIR, α-Amylase activity, α-Glucosidase activity, Molecular docking

## Abstract

In this study, 5-amino-nicotinic acid derivatives (**1**–**13**) have been designed and synthesized to evaluate their inhibitory potential against α-amylase and α-glucosidase enzymes. The synthesized compounds (**1**–**13**) exhibited promising α-amylase and α-glucosidase activities. IC_50_ values for α-amylase activity ranged between 12.17 ± 0.14 to 37.33 ± 0.02 µg/mL ± SEM while for α-glucosidase activity the IC_50_ values were ranged between 12.01 ± 0.09 to 38.01 ± 0.12 µg/mL ± SEM. In particular, compounds **2** and **4**–**8** demonstrated significant inhibitory activities against α-amylase and α-glucosidase and the inhibitory potential of these compounds was comparable to the standard acarbose (10.98 ± 0.03 and 10.79 ± 0.17 µg/mL ± SEM, respectively). In addition, the impact of substituent on the inhibitory potential of these compounds was assessed to establish structure activity relationships. Studies in molecular simulations were conducted to better comprehend the binding properties of the compounds. All the synthesized compounds were extensively characterized with modern spectroscopic methods including ^1^H-NMR, ^13^C–NMR, FTIR, HR-MS and elemental analysis.
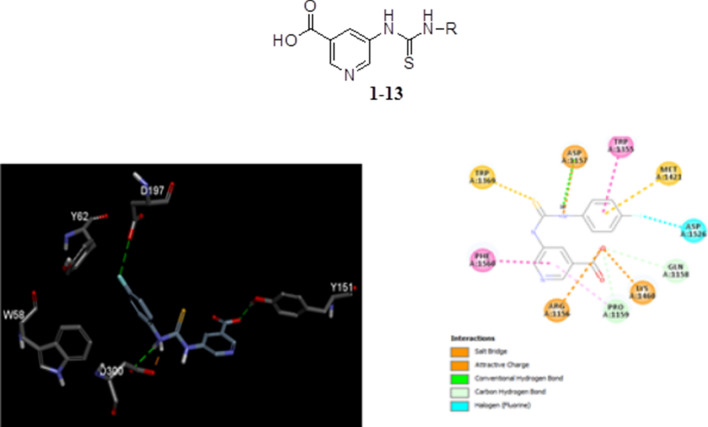

## Introduction

Diabetes, a metabolic disorder with colossal consequences is always at the forefront of medical discovery. The stats are rising alarmingly, and diabetes has become one of the top risk factors leading to death [1]. A weak or poor treatment regimen leads to associated complications such as stroke, heart arrest, organ failure, limb amputation, loss of vision and damage to nervous system, as well as increased risk of fetal death during poorly controlled gestational diabetes.

A simple delaying of the glucose absorption after food intake can play a key role towards the lifestyle and economic advantage of diabetic patients and their families, making Type II diabetes, unlike type I, considerably more curable and preventable. Therefore, an individualized treatment plan should be in place that suits the patients’ need better, with respect to age, and the presence of intrinsic and acquired resistance. Scientists have been researching for hypoglycemic agents with differing mechanisms. In order to cut the chase, the ideal hypoglycemic agent is considered the one which can address the issue of imbalance between blood sugar uptake and insulin secretion in the postprandial stage where two major saccharide hydrolyzing enzymes, α-amylase and α-glucosidase are the optimum targets for antidiabetic treatment [2]. Presently the dominant course of diabetes treatment involves insulin secretagogues and sensitizers, nevertheless, the uprising of disaccharide digesting enzyme inhibitors may be the future of controlling postprandial hyperglycemia [3].

As of yet, strong α-glucosidase inhibitors namely acarbose, miglitol, and voglibose are known for the control of postprandial hyperglycemia, but none of the above have competitive inhibiting potential against α-amylase. An ideal candidate that can competitively inhibit both α-amylase and α-glucosidase can synergistically reduce and control type II diabetes [4], resulting in improved lifestyle and increased life expectancy. Majority of the marketed disaccharide digesting enzyme inhibitors are of microbial origin but associated with side-effects [5, 6].

Diabetes also induces or increases the risk of many associated diseases [7] such as cardiovascular diseases and hypertension, and thus ideally requires use of multidrug therapeutic regimen for the concurrent diseases. The concomitant therapy in turn leads to complications and adverse effects [8–10]. The pancreatic inhibition of α-amylase and the action of α-glucosidase inhibitor in intestinal region can provide an additive effect in combating diabetes [11].

This research work is designed to identifying a new class of α-amylase inhibitors and α-glucosidase inhibitors that can not only control prevailing diabetes but also be of therapeutic significance in prediabetes stage of insulin resistance, where the onset of the diseases can either be entirely prevented or considerably delayed [12, 13]. It is well known that in clinical endocrinology, antidiabetics are preferably prescribed in combination with other therapeutic agents to control/suppress associated conditions. Considering the complications involved in achieving an effective combination of therapeutic agents, particularly for long-term use, it is ideal that broad spectrum carbohydrate digesting enzyme inhibitors are researched for optimum futuristic therapy for concurrent diseases.

For the most safe and versatile therapeutic solutions, we diverged into the plethora of naturally occurring medicinal compounds, specifically, nitrogen containing heterocyclic rings. Piperidine, pyridine and pyrimidine derivatives have been known to inhibit carbohydrate digesting enzymes, in addition to their anti-inflammatory, antibacterial and anticancer potencies [13–20]. In this study, derivatives of 5-amino-nicotinic acid are prepared, characterized and tested for α-amylase and α-glucosidase inhibiting potential.

## Results and discussion

### Chemistry

**Scheme 1:**
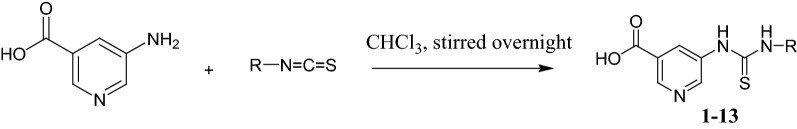
Synthesis of 5-amino-nicotinic acid thioureas derivatives (**1**–**13**)

Compounds (**1**–**13**) were prepared using 5-amino-nicotinic acid and phenyl isothiocyanates as depicted in the scheme 1. The structural confirmation of synthesized compounds (Scheme 1, Table 1) was achieved by ^1^H-NMR, ^13^C–NMR, HR-MS, elemental analysis and FTIR. All synthesized compounds structures are explored by identifying their respected chemical shift in proton and carbon NMR kindly see the experiment section for details.

**Table 1:** α-Amylase and α-glucosidase inhibition studies of compounds 1–13 with their CDOCKER interaction energy in kJ/mol

No.	R	α-Amylase inhibition (µg/mL ± SEM)	CDOCKER interaction energy in kJ/mol	α-Glucosidase inhibition (µg/mL ± SEM)	CDOCKER interaction energy in kJ/mol
1		28.89 ± 0.102	− 37.95	28.09 ± 0.09	− 38.30
2		12.91 ± 0.04	− 42.02	12.72 ± 0.12	− 42.16
3		28.84 ± 0.03	− 37.45	28.61 ± 0.11	− 37.87
4		12.17 ± 0.14	− 43.02	12.01 ± 0.09	− 43.23
5		13.57 ± 0.17	− 39.45	13.68 ± 0.36	− 39.05
6		13.01 ± 0.07	− 40.56	13.11 ± 0.15	− 39.42
7		12.91 ± 0.08	− 42.01	12.79 ± 0.17	− 42.31
8		13.04 ± 0.02	− 40.19	12.99 ± 0.09	− 41.03
9		26.53 ± 0.08	− 40.20	26.27 ± 0.18	− 40.95
10		26.7 ± 0.06	− 39.85	25.97 ± 0.19	− 40.82
11		26.94 ± 0.02	− 39.85	27.02 ± 0.11	− 40.03
12		37.33 ± 0.02	− 39.89	38.01 ± 0.12	− 39.48
13		36.65 ± 0.03	− 39.76	37.47 ± 0.13	− 39.38
Acarbose		10.98 ± 0.03	− 44.30	10.79 ± 0.17	− 44.79

### Antidiabetic studies

#### α-Amylase inhibitory activity

α-Amylase inhibitory potential of the compounds (**1**–**13**) was tested and results are summarized in Table 1. It was observed that synthesized compounds exhibited diverse α-amylase inhibitory activity. IC_50_ values for the compounds (**1**–**13**) ranged 12.17 ± 0.14 to 37.33 ± 0.02 µg/mL ± SEM. Compounds **2**, **4**, **5**, **6**, **7** and **8** (with IC_50_ values 12.91 ± 0.04, 12.17 ± 0.14, 13.57 ± 0.17, 13.01 ± 0.07, 12.91 ± 0.08 and 13.04 ± 0.02 µg/mL ± SEM, respectively) exhibited good activities for α-amylase inhibition and results were comparable with standard acarbose (10.98 ± 0.03 µg/mL ± SEM). Structure activity relationship of the compounds (**1**–**13**) is also discussed. The compound **4** was observed more potent with IC_50_ value 12.17 ± 0.14 µg/mL ± SEM, followed by compound **2** (12.91 ± 0.04 µg/mL ± SEM), and **7** (12.91 ± 0.08 µg/mL ± SEM). These results indicate that presence of halogens (F, Cl and Br) at the ring’s para position is more ideal for potential α-amylase inhibition as compare with other substituents. The compounds having methoxy (Compound **6**), trifluoro (compound **8**) and nitro group (compound **5**) also exhibited good α-amylase inhibition with IC_50_ value 13.01 ± 0.07, 13.04 ± 0.02 and 13.57 ± 0.17 µg/mL ± SEM, respectively. However, unsubstituted, di-substituted and ortho/meta substituents compounds didn’t show impressive α-amylase inhibition activity (**1**, **3** and **9**–**13** compounds) (Table 1).

#### α-Glucosidase inhibitory activity

Compounds (**1**–**13**) were tested for their potential α-glucosidase inhibitory action. IC_50_ values for α-glucosidase activity ranged between 12.01 ± 0.09 to 38.01 ± 0.12 µg/mL ± SEM. Compound **2** and **4**–**8** revealed significant activities for α-glucosidase inhibition with IC_50_ values 12.72 ± 0.12, 12.01 ± 0.09, 13.68 ± 0.36, 13.11 ± 0.15, 12.79 ± 0.17, and 12.99 ± 0.09 µg/mL ± SEM, respectively. Structure activity relationship of the compounds (**1**–**13**) is also conversed. The compounds **4**, **2** and **7** perceived significant α-glucosidase inhibition with IC_50_ values 12.01 ± 0.09, 12.72 ± 0.12, and 12.79 ± 0.17 µg/mL ± SEM, respectively. It is evident that the presence of halogens (F, Cl and Br) at the ring’s para position is more ideal for potential α-amylase inhibition as compare with other substituents. It is also noticed that compounds bearing trifluoro (compound **8**), methoxy (Compound **6**), and nitro group (compound **5**) also revealed α-amylase inhibition with IC_50_ value 12.99 ± 0.09, 13.11 ± 0.15 and 13.68 ± 0.36 µg/mL ± SEM, respectively. Conversely, unsubstituted, di-substituted and ortho/meta substituents compounds (**1**, **3**, and **9**–**13**) didn’t demonstrate remarkable α-glucosidase inhibition activity (Table 1).

### Molecular docking

#### α-Amylase docking study

**Fig. 1:**
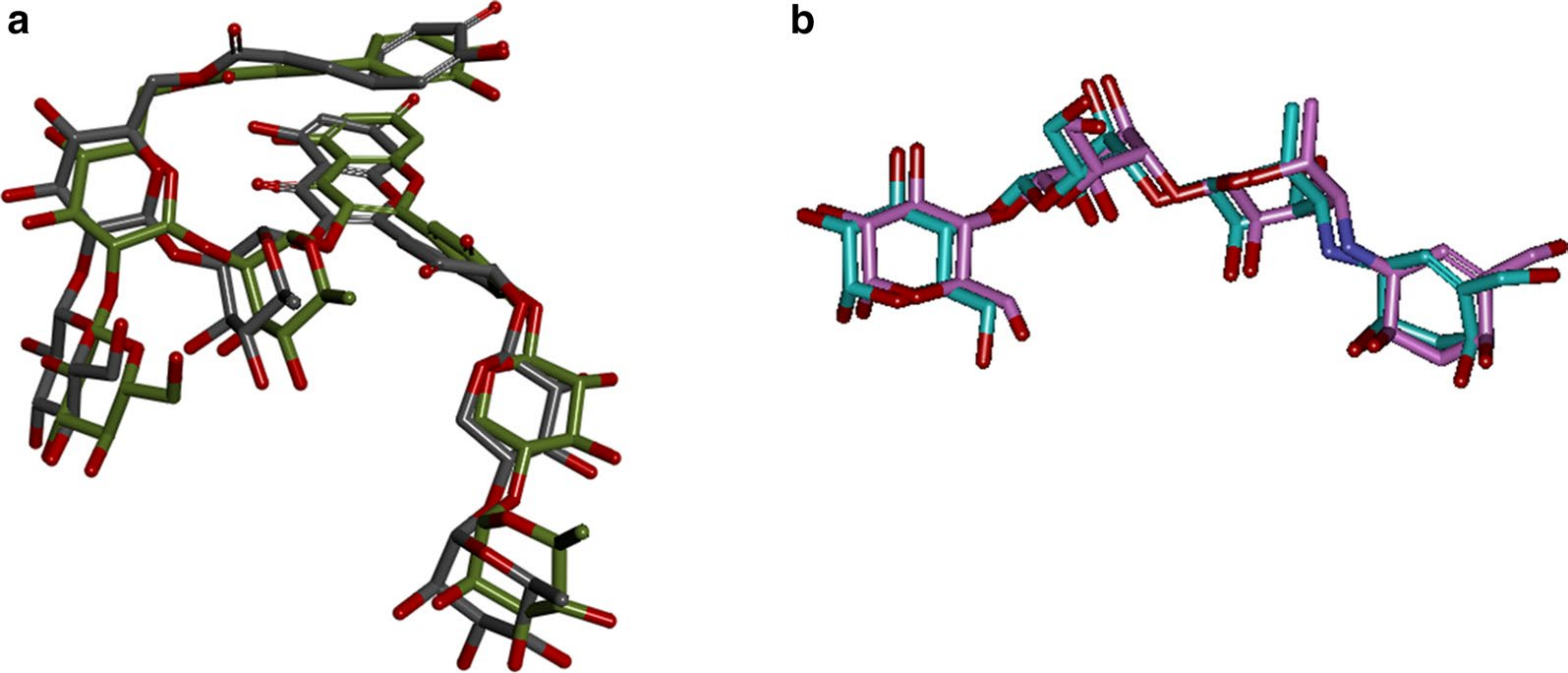
**a** Illustrates the superimposed the co-crystallized ligand in green (Montbretin A) with extracted Montbretin A (gray color) redocked to amylase crystal structure (pdb id: 4W93). **b** Illustrates the superimposed the co-crystallized ligand in blue (Acarbose) with extracted Acarbose (pink color) redocked to α-glucosidase crystal structure (pdb id: 3TOP)

Initially, the docking studies were validated by superimposing the co-crystallized ligand (Montbretin A) with extracted Montbretin A from crystal structure and redocked to amylase crystal structure (pdb id: 4W93). The calculated RMSD value between the X-ray Montbretin A (green) and redocked Montbretin A (gray) was 1.69 Å as shown in below Fig. 1a. Similarly, the superimposed the co-crystallized ligand in blue (Acarbose) with extracted Acarbose (pink color) redocked to α-glucosidase crystal structure (pdb id: 3TOP) and their RMSD value was 1.26 Å (Fig. 1b).

The CDOCKER interaction energy of 5-amino-nicotinic acid derivatives is reported in Table 1. The interaction energy − 42.01 kJ/mol and − 42.31 kJ/mol of the standard drug Montbretin A and acarbose for amylase and α-glucosidase was more stable than the 5-amino-nicotinic acid derivatives.

**Fig. 2:**
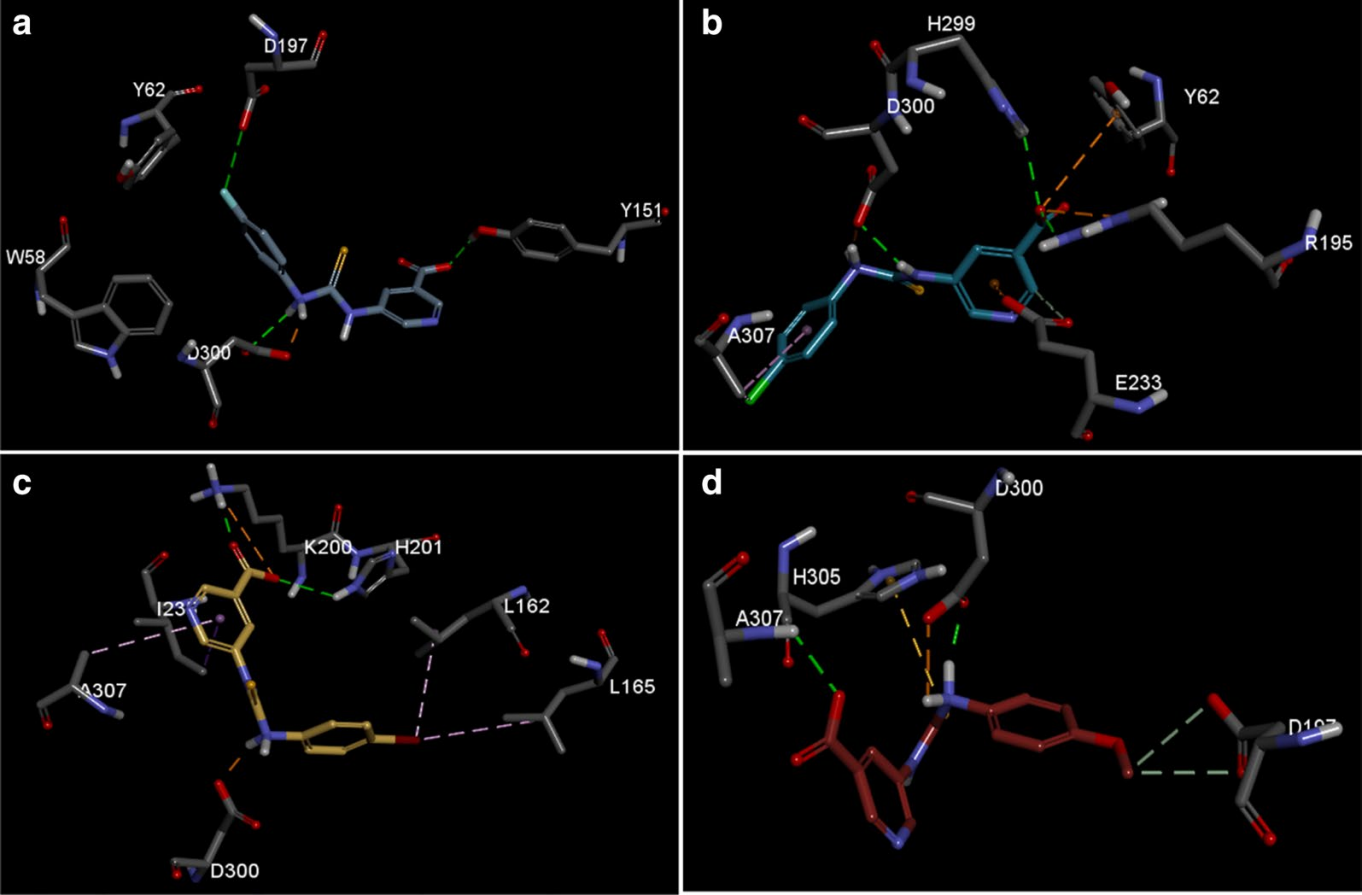
Illustrates the binding mode of 5-amino-nicotinic acid derivatives active compounds in α-amylase binding site. **a** Compound **4**, **b** compound **2**, **c** Compound **7** and **d** compound **6**. Key interaction types are represented in color code in each case (green: hydrogen bond, purple: alkyl contact, brown: pi stacking)

The docking studies show the binding model of the four highest active compounds in 5-amino-nicotinic acid thioureas derivatives, binding to the Montbretin A binding site of the alpha amylase. Figure 2a, illustrates the binding mode of the compound **2**, showing that the cholorbenzene interacts with D197 and forms hydrophobic contact with W58 and Y62. While, the amine group forms hydrogen bond and salt bridge with D300, respectively. The pyridine-3-carboxylate group contacts with D197 via hydrogen bonding.

In the study of the binding properties of the compound **2** (Fig. 2b) the chlorophenyl group forms both alkyl and pi-alkyl contact with A307. While two amine linkers form salt bridge and hydrogen bond contact with D300, respectively. The pyridine ring forms pi-anion contact and carbon hydrogen bond with E233. Finally, the carboxylate moiety forms hydrogen bond with sidechain of R195 and H299. The group also forms electrostatic pi-anion contact with Y62 and salt bridge with R195. Meanwhile, Fig. 2c shows the binding properties of the compound **7**, where the bromo group undergoes hydrophobic contact with L162 and L165. The amine moiety forms salt bridge with D300. The pyridine ring forms pi-sigma contact with I235 and pi-alkyl contact with A307. On the other hand, the carboxylate moiety forms hydrogen bond with sidechain of K200 and H201, it also forms electrostatic interaction with K200.

In the case of the fourth most active compound **6** the methoxy group forms carbon-based hydrogen bond with D197. The amine group forms both hydrogen bond and salt bridge with D300. This time the sulfur group forms pi-sulfur contact H305 and finally there is a hydrogen bond observed between the backbone nitrogen of A307 and carboxylate moiety (Fig. 2d).

Thus over all in this class of compound the presence of nonpolar groups as substituent was key responsible for establishing the notable interaction between 5-amino-nicotinic acid derivatives and the key residues on the active site of the alpha amylase, eventually reflecting in the biological activity profile.

#### α-Glucosidase docking study

**Fig. 3:**
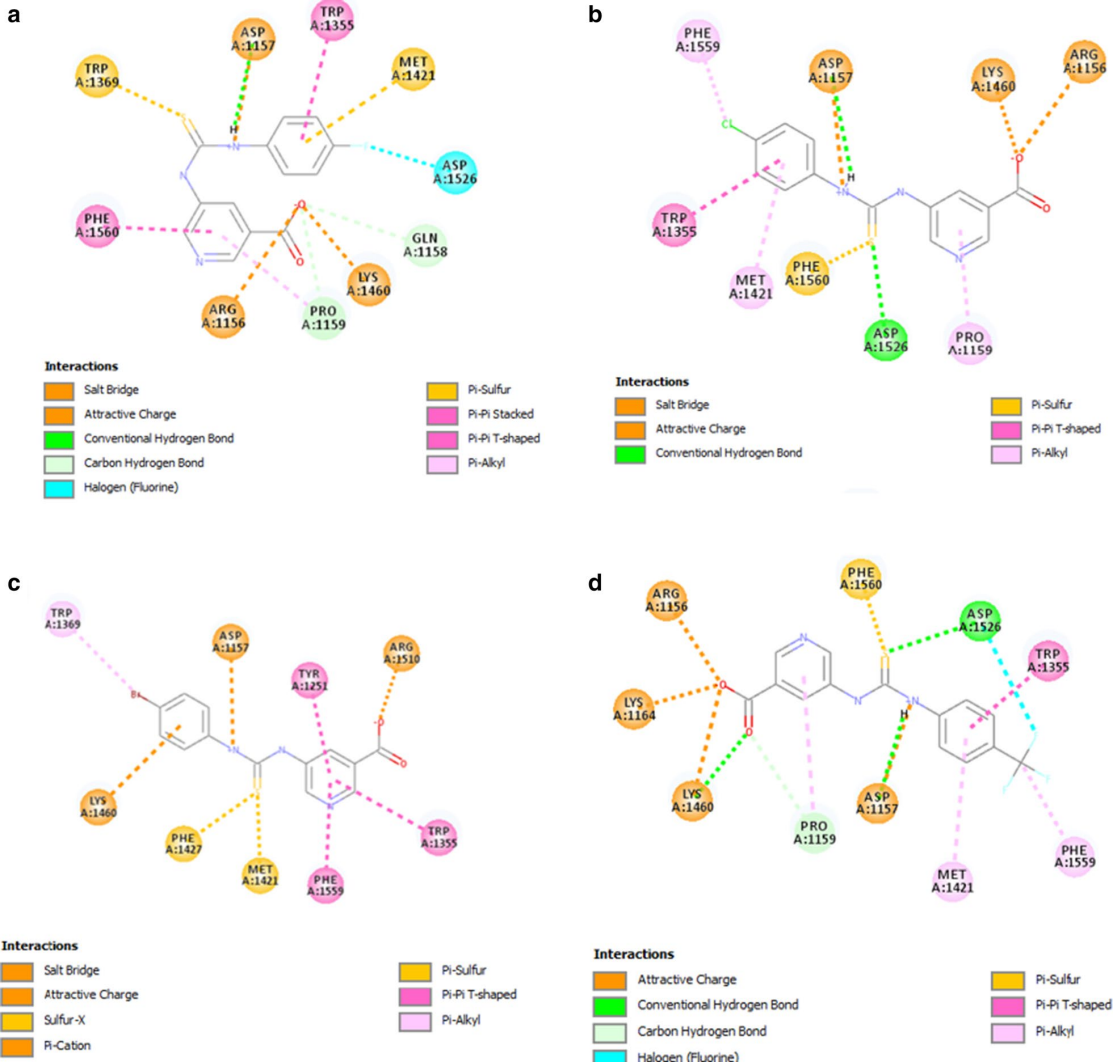
Illustrates the binding mode of 5-amino-nicotinic acid derivatives active compounds in α-glucosidase. **a** Compound **4**, **b** compound **2**, **c** Compound **7** and **d** compound **8**. Key interaction types are represented in color code in each case

Binding mode of compound **4** is shown in Fig. 3a, where the fluoro group of the fluorophenyl ring forms halogen contact with Asp1526 and the ring of the system, as well as form pi-sulfur interaction in conjunction with Met1421 and pi–pi T-shaped stacking with Trp1355. The NH group linking the carbamothioyl and amino group forms hydrogen bond with Asp1157 and attractive charge interaction with the same group. The thiol group also forms pi-sulfur contact with Trp1369. On the other end the pyridine-3-carboxylate forms pi–pi stacking with Phe1560 and carbon hydrogen bonding with Pro1159. The carboxylate oxygen forms salt bridge with Arg1156 and Lys1460.

Figure 3b shows the binding mode of compound **2**, the chloro group of the chlorophenyl forms pi-alkyl interaction with Phe1159 and the phenyl ring forms both pi–pi and pi-T-shaped interaction with Met1421 and Trp1355. Likewise in compound **4** the NH group forms both attractive and hydrogen bond interaction with Asp1157. The thiol group forms pi-sulfur contact with Phe1560 and hydrogen bond with Asp1526. The pyridine-3-carboxylate moiety forms pi-alkyl contact with Pro1159 and the carboxyl group forms salt bridge with Arg1156 and Lys1460, respectively.

In the case of the compound **7**, which contains a bromophenyl moiety, pi-alkyl interaction was observed with Trp1369 and pi-cation interaction between the ring of the bromophenyl group and Lys1460. The NH of the linker forms attractive charge with Asp1157 and on the other hand the thiol group forms pi-sulfur interaction with Phe1427 and Met1421. Likewise, in the other two above active compounds the pyridine-3-carboxylate moiety forms pi–pi interaction with Tyr1251, Trp1355 and Phe1559, respectively. In addition, the carboxylate group forms salt bridge with Arg1510 (Fig. 3c).

Binding mode of compound **8** is shown in Fig. 3d. The ring of trifluoromethylphenyl forms both pi-alkyl and pi–pi T-shaped stacking with Met1421 and Trp1355, while the fluoro group forms halogen contact and the methyl forms pi-alkyl interaction, correspondingly. Likewise, the NH group linker forms both hydrogen bond and attractive interaction with Asp1157. The thiol group connector forms pi-sulfur contact and hydrogen bond contact with Phe1560 and Asp1526, respectively. Finally, the pyridine-3-carboxylate moiety forms pi-alkyl interaction with Pro1159 and the carboxyl group oxygens forms attractive charge interaction with Arg1156, Lys1164 and Lys1460. There is also a hydrogen bond between the oxygen and the Lys1460.

Thus, to conclude, we could say that the alkyl group present at the para position was mostly preferred in this class of compound, the activity was determined either by pi-alkyl contact or halogen contact with the core residues of the active site of the α glucosidase.

## Conclusion

5-Amino-nicotinic acid derivatives (**1–13**) have been synthesized successfully and assessed for their action against α-amylase and α-glucosidase. Compounds **2**, and **4–8** revealed major activities for α-amylase and α-glucosidase inhibition. This study identifies new class of compounds as α-amylase and α-glucosidase inhibitors.

## Experimental

### Materials and methods

All chemicals and enzymes (α-amylase and α-glucosidase enzyme) used in this study were purchase from Sigma Aldrich. Avance Bruker 500 MHz has been used for carrying out ^1^H-NMR and ^13^C–NMR. FTIR (PerkinElmer) was employed to study the functional groups of the compounds. HR-MS were determined on Agilent 6330 Ion Trap using positive/negative mode. Elemental analysis was performed using PerkinElmer instrument. Melting point was recorded on Stuart (SMP-10) melting point apparatus. Pre-coated silica gel aluminum foils (Germany) were utilized for execution of thin layer chromatography (TLC). UV lamp was used for visualizing chromatograms.

### *α*-Amylase inhibitory activity

The inhibition of *α*-amylase was established by the methods described earlier [21, 22]. Incubation of 500 µL of test sample (1–100 µg/mL) along with 500 µL of *α*-amylase (0.5 mg/mL in phosphate buffer; 0.2 mM maintained at pH 6.9) was carried out for 10 min at 25 °C. After pre-incubation, 1% starch solution was added (500 μL, in 0.2 mM phosphate buffer maintained at pH 6.9) and incubated at 25 °C for another 10 min. The reaction was brought to arrest by using 1 mL of di-nitro-salicylic acid color reagent. The tubes were afterwards refluxed for 5 min and then cooled at ambient temperature. Resulting solutions, after diluted with 10 mL of distilled water, were analyzed at 540 nm by recording absorbance [23]. Acarbose was used as the standard drug.

The percentage of inhibition was estimated by employing the formula;

$$ \%_{\text{Inhibition}} = \left( {{\text{A}}_{\text{Control}} - {\text{A}}_{\text{Sample}} } \right)/{\text{A}}_{\text{Control}} \times 100. $$

The optimum concentration needed to hydrolyze the *α*-amylase by 50% (IC_50_ values) was computed via non-linear regression plot of % inhibition at x axis and concentrations at y axis, with the help of Graph Pad Prism Software (Ver. 5).

### α-Glucosidase activity

The inhibition of α-glucosidase was established with the help of the modified version of the published technique [24]. α-glucosidase solution was prepared by dissolving 1 mg in 100 mL phosphate buffer (pH 6.8) comprising of 200 mg bovine serum albumin. 10 μL of sample at variable concentrations (1 to 100 μg/mL) was pre-mixed with 490 μL phosphate buffer (pH 6.8), and to this reaction mixture was added 250 μL of 5 mM p-nitrophenyl α-d-glucopyranoside and preincubated at 37 °C. After 5 min, addition of 250 μL α-glucosidase (0.15 unit/mL) was done with further incubation at 37 °C for 15 min. The inhibition was concluded by adding 2000 μL of Na_2_CO_3_ (200 mM). α-Glucosidase activity was computed at 400 nm on Shimadzu 265 UV–Vis spectrophotometer (Japan) by computing the amount of *p*-nitrophenol released from p-NPG, using acarbose as positive control. The optimum concentration needed to hydrolyze 50% of α-glucosidase was defined as the IC_50_ value.

The percentage of inhibition was estimated by employing the formula;

$$ \%_{\text{Inhibition}} = \left( {{\text{A}}_{\text{Control}} - {\text{A}}_{\text{Sample}} } \right)/{\text{A}}_{\text{Control}} \times 100. $$

### Statistical analysis

The concentration required to inhibit the α-amylase by 50% (IC_50_ values) were computed via non-linear regression plot between percentage inhibition at the x axis and concentrations at y axis, with the help of Graph Pad Prism Software (Ver. 5).

### Computational docking methodology

#### Computational docking studies of α-amylase and α-glucosidase

With the aim of revealing the binding properties of the all the 5-amino-nicotinic acid derivatives in α-amylase (pdb id: 4w93) [25] and in α-glucosidase (pdb id: 3top) [26], molecular docking studies were done using CDOCKER implemented in Discovery studio.CDOCKER is a grid-based method of molecular docking that make use of CHARMM force field [27]. The **Montbretin A** binding site in α-amylase crystal structure was defined as binding site for docking the compounds. Similarly the acarbose binding site in α-glucosidase crystal structure was defied as the binding site to dock the compounds. Prior to docking both enzymes and the 5-amino-nicotinic acid derivatives were structurally optimized by adding hydrogen and atom valencies were satisfied so that atoms are properly typed. After the binding site sphere was defined, docking calculation was subsequently done. Top 10 binding pose were opted for prediction and results were analysed using Discovery studio visualizer.

### General procedure for synthesis of 5-amino-nicotinic acid derivatives

1 mmol of 5-amino-nicotinic acid was weighed and transfer into 50 mL round bottomed flask, then 1.2 mmol of phenyl isothiocyanate (Table 1) was added, followed by the addition of 10 mL of chloroform. The reaction mixture was left overnight with stirring and was monitored by TLC. After the reaction was completed, the product was transferred into beakers and evaporated at room temperature. Diethyl ether was used to wash the solid product.

#### 5-(3-Phenylthioureido)pyridine-3-carboxylic acid (**1**)

Yield: 76%; M.p.: > 300 °C; FTIR (ATR, cm^−1^): 3332 (N–H), 3190 (Ar–CH), 1654 (C=O), 1586 (C–N), 1488 (C=C),1330 (C=S); ^1^HNMR (500 MHz, DMSO-*d*_*6*_): *δ* 12.16 (s, 1H, NH), 11.50 (s, 1H, NH), 8.86 (s, 1H, H-2), 8.62 (s, 1H, H-6), 7.69 (s, 1H, H-4), 7.06–6.94 (m, 5H, H-2′to H-5′), 3.60 (br. s, 1H, OH); ^13^CNMR (125 MHz, DMSO-*d*_*6*_): *δ* 179.6 (C=S), 169.0 (C=O), 141.2 (C2), 140.5 (C6), 137.0 (C1′), 134.2 (C5), 129.0 (C3′) 129.0 (C5′), 128.4 (C3), 126.4 (C2′), 126.4 (C6′), 124.3 (C4′), 122.8 (C4); HR-MS for C_13_H_11_N_3_O_2_S calculated 273.0572 and found 273.0549; Anal. calcd. for C_13_H_11_N_3_O_2_S: C, 57.13; H, 4.06; N, 15.37;O, 11.71; S, 11.73; found: C, 57.11; H, 4.05; N, 15.36; O, 11.70; S, 11.72.

#### 5-(3-(4-Chlorophenyl)thioureido)pyridine-3-carboxylic acid (**2**)

Yield: 72%; M.p.: > 300 °C; FTIR (ATR, cm^−1^): 3334 (N–H), 3191 (Ar–CH), 1655 (C=O), 1587 (C–N), 1469 (C=C), 1331(C=S), 785(C–Cl); ^1^HNMR (500 MHz, DMSO-*d*_*6*_): *δ* 11.50 (s, 1H, NH), 11.30 (s, 1H, NH), 8.85 (s, 1H, H-2), 8.60 (s, 1H, H-6), 7.62 (s, 1H, H-4), 7.03 (d, *J *= 8.0 Hz, 2H, H-2′, H-6′), 6.40 (d, *J *= 8.0 Hz, H-3′, H-5′), 3.58 (br. s, 1H, OH); ^13^CNMR (125 MHz, DMSO-*d*_*6*_): *δ* 179.5 (C=S), 169.0 (C=O), 141.1 (C2), 140.0 (C6), 135.0 (C1′), 134.3 (C5), 130.1(C4′), 129.0 (C3′), 129.0 (C5′), 128.8 (C3), 127.5 (C 2′), 127.5 (C6′), 123.3 (C4); HR-MS for C_13_H_10_ClN_3_O_2_S calculated 307.0182 and found 307.0171; Anal. calcd. for C_13_H_10_ClN_3_O_2_S: C, 50.73; H, 3.28; N, 13.65; O, 10.40; S, 10.42. Found: C, 50.72; H, 3.26; N, 13.64; O, 10.39; S, 10.41.

#### 5-(3-p-Tolylthioureido)pyridine-3-carboxylic acid (**3**)

Yield: 79%; M.p.: > 300 °C; FTIR (ATR, cm^−1^): 3334 (N–H), 3191 (Ar–CH), 1655 (C=O), 1586 (C–N), 1468 (C=C), 1331 (C=S). HR-MS; ^1^HNMR (500 MHz, DMSO-*d*_*6*_): *δ* 11.80 (s, 1H, NH), 11.10 (s, 1H, NH), 8.87 (s, 1H, H-2), 8.61(s, 1H, H-6), 7.61 (s, 1H, H-4), 6.80 (d, *J *= 8.5 Hz, 2H, H-3′, H-5′), 6.32 (d, *J *= 8.5 Hz, 2H, H-2′, H-6′), 3.62 (br. s, 1H, OH), 2.46 (s, 3H, CH_3_); ^13^CNMR (125 MHz, DMSO-*d*_*6*_): *δ* 179.3 (C=S), 169.3 (C=O), 141.2 (C2), 140.2 (C6), 134.3 (C4′), 134.2 (C1′), 134.0 (C5), 129.2 (C3′), 129.2 (C5′), 128.5 (C3), 127.0 (C2′), 127.0 (C6′), 123.1 (C4), 24.2 (CH_3_); for C_14_H_13_N_3_O_2_S calculated 287.0728 and found 287.0717; Anal. calcd. for C_14_H_13_N_3_O_2_S: C, 58.52; H, 4.56; N, 14.62; O, 11.14; S, 11.16 found: C, 58.50; H, 4.55; N, 14.60; O, 11.13; S, 11.15.

#### 5-(3-(4-Fluorophenyl)thioureido)pyridine-3-carboxylic acid (**4**)

Yield: 75%; M.p.: > 300 °C; FTIR (ATR, cm^−1^): 3334 (N–H), 3191 (Ar–CH), 1655 (C=O), 1586 (C–N), 1469(C=C), 1331 (C=S), 785 (C-F); ^1^HNMR (500 MHz, DMSO-*d*_*6*_): *δ* 11.50 (s, 1H, NH), 11.15 (s, 1H, NH), 8.86 (s, 1H, H-2), 8.62 (s, 1H, H-6), 7.62 (s, 1H, H-4), 6.72 (d, *J *= 7.5 Hz, 2H, H-3′, H-5′), 6.40 (t, *J *= 8.5 Hz, 2H, H-2′, H-6′), 3.62 (br. s, 1H, OH); ^13^CNMR (125 MHz, DMSO-*d*_*6*_): *δ* 179.7 (C=S), 169.0 (C=O), 158.5 (C4′), 140.5 (C2), 140.0 (C6), 134.3 (C5), 132.1 (C1′), 128.8 (C2′), 128.8 (C6′), 128.3 (C3), 115.6 (C3′), 115.6 (C5′), 123.2 (C4); HR-MS for C_13_H_10_FN_3_O_2_S calculated 291.0478 and found 291.0463; Anal. calcd. for C_13_H_10_FN_3_O_2_S: C, 53.60; H, 3.46; N, 14.42; O, 10.98; S, 11.01; found: C, 53.59; H, 3.44; N, 14.41; O, 10.97; S, 11.01.

#### 5-(3-(4-Nitrophenyl) thioureido) pyridine-3-carboxylic acid (**5**)

Yield: 69%; M.p.: > 300 °C; FTIR (ATR, cm^−1^): 3333 (N–H), 3190 (Ar–CH), 1654 (C=O), 1586 (C–N), 1468(C=C), 1330 (C=S);^1^HNMR (500 MHz, DMSO-*d*_*6*_): *δ*11.42 (s, 1H, NH), 11.02 (s, 1H, NH), 8.86 (s, 1H, H-2), 8.60 (s, 1H, H-6), 7.90 (d, *J *= 8.0 Hz, 2H, H-3′, H-5′), 7.58 (s, 1H, H-4), 6.68 (d, *J *= 8.0 Hz, 2H, H-2′, H-6′), 3.60 (br. s, 1H, OH); ^13^CNMR (125 MHz, DMSO-*d*_*6*_): *δ* 179.6 (C=S), 169.2 (C=O), 144.5 (C4′), 143.4 (C1′), 140.5 (C2), 140.0 (C6), 133.9 (C5), 128.9 (C3), 127.2 (C2′), 127.2 (C6′), 121.2 (C3′), 121.2 (C5′), 123.1 (C4);HR-MS for C_13_H_10_N_4_O_4_S calculated 318.0423 and found 318.0416; Anal. calcd. for C_13_H_10_N_4_O_4_S: C, 49.05; H, 3.17; N, 17.60; O, 20.11; S, 10.07; found: C, 49.03; H, 3.16; N, 17.58; O, 20.10; S, 10.06.

#### 5-(3-(4-Methoxyphenyl) thioureido) pyridine-3-carboxylic acid (**6**)

Yield: 77%; M.p.: > 300 °C; FTIR (ATR, cm^−1^): 3333 (N–H), 3190 (Ar–CH), 1654 (C=O), 1586 (C–N), 1468(C=C), 1330(C=S),1089(Ar–O–C); ^1^HNMR (500 MHz, DMSO-*d*_*6*_): *δ* 11.42 (s, 1H, NH), 11.05 (s, 1H, NH), 8.86 (s, 1H, H-2), 8.60 (s, 1H, H-6), 7.59 (s, 1H, H-4), 6.52 (d, *J *= 8.0 Hz, 2H, H-3′, H-5′), 6.37 (d, *J *= 8.0 Hz, 2H, H-2′, H-6′), 3.80 (s, 3H, OCH_3_) 3.60 (br. s, 1H, OH); ^13^CNMR (125 MHz, DMSO-*d*_*6*_): *δ* 179.6 (C=S), 169.0 (C=O), 156.4 (C4′), 140.5 (C2), 140.0 (C6), 134.2 (C5), 129.2 (C1′), 128.9 (C3), 127.3 (C2′), 127.3 (C6′), 123.1 (C4), 114.2 (C3′), 114.2 (C5′), 55.6 (CH_3_); HR-MS for C_14_H_13_N_3_O_3_S calculated 303.0678 and found 303.0667; Anal. calcd. for C_14_H_13_N_3_O_3_S: C, 55.43; H, 4.32; N, 13.85; O, 15.82; S, 10.57; found: C, 55.42; H, 4.31; N, 13.83; O, 15.81; S, 10.56.

#### 5-(3-(4-Bromophenyl) thioureido) pyridine-3-carboxylic acid (**7**)

Yield: 79%; M.p.: > 300 °C; FTIR (ATR, cm^−1^): 3333 (N–H), 3190 (Ar–CH), 1654 (C=O), 1586 (C–N), 1468 (C=C), 1331(C=S) 785(C–Br);^1^HNMR (500 MHz, DMSO-*d*_*6*_): *δ*11.70 (s, 1H, NH), 11.30 (s, 1H, NH), 8.80 (s, 1H, H-2), 8.62 (s, 1H, H-6), 7.70 (s, 1H, H-4), 7.15 (d, *J *= 8.0 Hz, 2H, H-3′, H-5′), 6.38 (d, *J *= 8.0 Hz, 2H, H-2′, H-6′), 3.64 (br. s, 1H, OH); ^13^CNMR (125 MHz, DMSO-*d*_*6*_): *δ* 179.6 (C=S), 169.3 (C=O), 140.3 (C2), 140.1 (C6), 136.0 (C1′), 134.2 (C5), 132.1 (C3′), 132.1 (C5′), 128.6 (C3), 128.5 (C2′), 128.5 (C6′), 123.1 (C4), 119.2 (C4′); HR-MSfor C_13_H_10_BrN_3_O_2_S calculated 350.9677 and found 350.9653; Anal. calcd. for C_13_H_10_BrN_3_O_2_S: C, 44.33; H, 2.86; N, 11.93; O, 9.09; S, 9.10; found: C, 44.32; H, 2.84; N, 11.92; O, 9.08; S, 9.08.

#### 5-(3-(4-(Trifluoromethyl)phenyl) thioureido) pyridine-3-carboxylic acid (**8**)

Yield: 72%; M.p.: > 300 °C; FTIR (ATR, cm^−1^): 3333 (N–H), 3190 (Ar–CH), 1654 (C=O), 1586 (C–N), 1468(C=C), 1330(C=S),785(C-F); ^1^HNMR (500 MHz, DMSO-*d*_*6*_): *δ* 11.62 (s, 1H, NH), 11.32 (s, 1H, NH), 8.72 (s, 1H, H-2), 8.65 (s, 1H, H-6), 7.67 (s, 1H, H-4), 7.15 (d, *J *= 8.0 Hz, 2H, H-3′, H-5′), 6.38 (d, *J *= 8.0 Hz, 2H, H-2′, H-6′), 3.68 (br. s, 1H, OH); ^13^CNMR (125 MHz, DMSO-*d*_*6*_): *δ* 179.6 (C=S), 169.3 (C=O), 140.6 (C2), 140.1 (C6), 140.0 (C1′), 134.2 (C5), 128.8 (C3), 127.3 (CF_3_), 126.7 (C2′), 126.7 (C6′), 125.5 (C3′), 125.5 (C5′), 124.1 (C4′), 123.1 (C4); HR-MS for C_14_H_10_F_3_N_3_O_2_S calculated 341.0446 and found 341.0432; Anal. calcd. for C_14_H_10_F_3_N_3_O_2_S: C, 49.27; H, 2.95; F, 16.70; N, 12.31; O, 9.38; S, 9.39; found: C, 49.26; H, 2.93; F, 16.69; N, 12.30; O, 9.37; S, 9.37.

#### 5-(3-(3-Chloro-4-methylphenyl) thioureido) pyridine-3-carboxylic acid (**9**)

Yield: 68%; M.p.: > 300 °C; FTIR (ATR, cm^−1^): 3333 (N–H), 3190 (Ar–CH), 1654 (C=O), 1586 (C–N), 1468(C=C), 1330(C=S), 785(C–Cl); ^1^HNMR (500 MHz, DMSO-*d*_*6*_): *δ* 11.43 (s, 1H, NH), 11.23 (s, 1H, NH), 8.72 (s, 1H, H-2), 8.69 (s, 1H, H-6), 7.61 (s, 1H, H-4), 6.74 (d, *J *= 7.5 Hz, 1H, H-5′), 6.37 (s, 1H, H-2′), 6.21 (d, *J *= 8.0 Hz, 1H, H-6′), 3.64 (br. s, 1H, OH), 2.38 (s, 3H, CH_3_); ^13^CNMR (125 MHz, DMSO-*d*_*6*_): *δ* 179.8 (C=S), 169.5 (C=O), 140.5 (C2), 140.2 (C6), 135.6 (C1′), 134.6 (C3′), 134.1 (C5), 132.1 (C4′), 130.5 (C5′), 128.9 (C3), 126.7 (C2′), 124.7 (C6′), 123.1 (C4), 15.6 (CH_3_); HR-MS for C_14_H_12_ClN_3_O_2_S calculated 321.0339 and found 321.0327; Anal. calcd. for C_14_H_12_ClN_3_O_2_S: C, 52.26; H, 3.76; N, 13.06; O, 9.94; S, 9.96; found: C, 52.25; H, 3.75; N, 13.04; O, 9.92; S, 9.95.

#### 5-(3-(2-Fluorophenyl) thioureido) pyridine-3-carboxylic acid (**10**)

Yield: 79%; M.p.: > 300 °C; FTIR (ATR, cm^−1^): 3333 (N–H), 3191 (Ar–CH), 1655 (C=O), 1586 (C–N), 1469(C=C), 1331(C=S), 785(C-F); ^1^HNMR (500 MHz, DMSO-*d*_*6*_): *δ* 11.96 (s, 1H, NH), 11.38 (s, 1H, NH), 8.74 (s, 1H, H-2), 8.65 (s, 1H, H-6), 7.67 (s, 1H, H-4), 6.75–6.70 (m, 2H, H-2′, H-5′), 6.63–6.60 (m,1H, H-4′), 6.41 (d, *J *= 7.0 Hz, 1H, H-6′), 3.66 (br. s, 1H, OH); ^13^CNMR (125 MHz, DMSO-*d*_*6*_): *δ* 179.8 (C=S), 169.4 (C=O), 167.6 (C2′), 140.3 (C2), 140.1 (C6), 134.1 (C5), 128.8 (C3), 128.2 (C6′), 126.4 (C4′), 124.6 (C5′), 123.3 (C4), 120.3 (C1′), 115.6 (C3); HR-MS for C_13_H_10_FN_3_O_2_S calculated 291.0478 and found 291.0464; Anal. calcd. for C_13_H_10_FN_3_O_2_S: C, 53.60; H, 3.46; N, 14.42; O, 10.98; S, 11.01; found: C, 53.58; H, 3.44; N, 14.41; O, 10.97; S, 11.01.

#### 5-(3-(3-Fluorophenyl) thioureido) pyridine-3-carboxylic acid (**11**)

Yield: 75%; M.p.: > 300 °C; FTIR (ATR, cm^−1^): 3331 (N–H), 3191 (Ar–CH), 1655 (C=O), 1587 (C–N), 1468(C=C), 1332(C=S), 785(C-F); ^1^HNMR (500 MHz, DMSO-*d*_*6*_): *δ* 11.92 (s, 1H, NH), 11.34 (s, 1H, NH), 8.75 (s, 1H, H-2), 8.64 (s, 1H, H-6), 7.65 (s,1H, H-4), 6.96–6.92 (m, 1H, H-5′), 6.35–6.30 (m, 2H, H-4′, H-6′), 6.21 (d, *J *= 7.5 Hz, 1H, H-1′), 3.68 (br. s, 1H, OH); ^13^CNMR (125 MHz, DMSO-*d*_*6*_): *δ* 179.8 (C=S), 169.4 (C=O), 163.1 (C3′), 140.3 (C2), 140.1 (C6), 138.6 (C1′), 134.1 (C5), 130.5 (C5′), 128.4 (C3), 123.1 (C4), 122.0 (C6′), 115.4 (C2′), 111.3 (C4′); HR-MS for C_13_H_10_FN_3_O_2_S calculated 291.0478 and found 291.0458; Anal. calcd. for C_13_H_10_FN_3_O_2_S: C, 53.60; H, 3.46; N, 14.42; O, 10.98; S, 11.01; found: C, 53.59; H, 3.45 N, 14.41; O, 10.97; S, 11.01.

#### 5-(3-(2-Bromophenyl) thioureido) pyridine-3-carboxylic acid (**12**)

Yield: 71; M.p.: > 300 °C; FTIR (ATR, cm^−1^): 3333 (N–H), 3191 (Ar–CH), 1655 (C=O), 1586 (C–N), 1468(C=C), 1331(C=S), 785(C–Br); ^1^HNMR (500 MHz, DMSO-*d*_*6*_): *δ* 11.72 (s, 1H, NH), 11.31 (s, 1H, NH), 8.70 (s, 1H, H-2), 8.61 (s, 1H, H-6), 7.63 (s,1H, H-4), 7.03 (t, *J *= 7.0 Hz, 1H, H-3), 6.98–6.95 (m, 1H, H-5), 6.45–6.40 (m, 2H, H-4, H-6), 3.64 (br. s, 1H, OH);^13^CNMR (125 MHz, DMSO-*d*_*6*_): *δ* 179.5 (C=S), 169.3 (C=O), 140.3 (C2), 140.1 (C6), 137.4 (C1′), 134.3 (C5), 132.2 (C3′), 128.9 (C3), 128.6 (C6′), 128.0 (C5′), 127.4 (C2′), 127.1 (C4′), 123.4 (C4); HR-MS for C_13_H_10_BrN_3_O_2_S calculated 350.9677 and found 350.9677; Anal. calcd. for C_13_H_11_N_3_O_2_S: C, 44.33; H, 2.86; N, 11.93; O, 9.09; S, 9.10; found: C, 44.31; H, 2.85; N, 11.91; O, 9.07; S, 9.09.

#### 5-(3-(3-Bromophenyl) thioureido) pyridine-3-carboxylic acid (**13**)

Yield: 73%; M.p.: > 300 °C; FTIR (ATR, cm^−1^): 3337 (N–H), 3191 (Ar–CH), 1655 (C=O), 1586 (C–N), 1468(C=C), 1331(C=S), 785(C–Br); ^1^HNMR (500 MHz, DMSO-*d*_*6*_): *δ* 11.78 (s, 1H, NH), 11.30 (s, 1H, NH), 8.72 (s, 1H, H-2), 8.60 (s, 1H, H-6), 7.62 (s, 1H, H-4), 6.88–6.85 (m, 1H, H-5′), 6.76–6.73 (m, 1H, H-4′), 6.62 (d, *J *= 2.0 Hz, 1H, H-2′), 6.42 (t, *J *= 7.5 Hz, 1H, H-6′), 3.66 (br. s, 1H, OH);^13^CNMR (125 MHz, DMSO-*d*_*6*_): *δ* 179.5 (C=S), 169.3 (C=O), 140.3 (C2), 140.1 (C6), 139.4 (C1′), 134.3 (C5), 131.2 (C5′), 128.9 (C3), 127.5 (C4′), 125.7 (C6′), 125.3 (C2′), 123.6 (C3′), 123.4 (C4); HR-MS for C_13_H_10_BrN_3_O_2_S calculated 447.0927 and found 447.0911; Anal. calcd. for C_13_H_10_BrN_3_O_2_S: C, 44.33; H, 2.86; 11.93; O, 9.09; S, 9.10; found: C, 44.32; H, 2.85; N, 11.92; O, 9.08; S, 9.08.

## Data Availability

The datasets used and/or analysed during the current study will be available from the corresponding author on reasonable request.

## Abbreviations

NMR: nuclear magnetic resonance spectroscopy
FTIR: Fourier-transform infrared spectroscopy
ATR: attenuated total reflection
HR-MS: high resolution mass spectrometry
TLC: thin layer chromatography
p-NPG: 4-nitrophenyl-α-d-glucopyranoside
